# Immunostaining and Ciliary Movement Analysis in Primary Ciliary Dyskinesia With a Homozygous Deletion Involving Exons 1–4 of *DRC1*: A Case Report

**DOI:** 10.1002/rcr2.70424

**Published:** 2025-12-01

**Authors:** Atsushi Kurokawa, Mitsuko Kondo, Mayoko Tsuji, Yuno Shiota, Azusa Miyoshi, Fumi Miyata, Keiko Kan‐o, Tomohiro Akaba, Ken Arimura, Osamitsu Yagi, Kaname Nakatani, Makoto Ikejiri, Kazuhiko Takeuchi, Etsuko Tagaya

**Affiliations:** ^1^ Department of Respiratory Medicine Tokyo Women's Medical University Tokyo Japan; ^2^ Department of Genomic Medicine Mie University Hospital Tsu Japan; ^3^ Department of Central Laboratories Mie University Hospital Tsu Japan; ^4^ Department of Otorhinolaryngology, Head & Neck Surgery Mie University Graduate School of Medicine Tsu Japan; ^5^ Department of Otorhinolaryngology Matsusaka Central General Hospital Matsusaka Japan

**Keywords:** cilia, diffuse panbronchiolitis, dynein regulatory complex subunit 1, immunofluorescence, primary ciliary dyskinesia

## Abstract

Primary ciliary dyskinesia (PCD) presents various clinical manifestations, including bronchiectasis, chronic sinusitis, situs inversus, and infertility. PCD is diagnosed through multiple methods, including transmission electron microscopy (TEM), high‐speed video microscopy analysis (HSVA), immunofluorescence (IF), and genetic testing. Recent reports show that a homozygous deletion involving exons 1–4 of *DRC1* is common in Japanese patients with PCD. We report the case of a 29‐year‐old male without situs inversus previously diagnosed with diffuse panbronchiolitis, who underwent PCD testing. TEM appeared almost normal, and HSVA showed motile cilia with reduced amplitude. Genetic testing revealed a homozygous deletion involving exons 1–4 of *DRC1*. Due to lack of a suitable DRC1 antibody for IF, we used the DRC3 antibody as in previous studies. IF showed the absence of DRC3 in the cilia, suggesting a *DRC1* deletion affects DRC3 localization in the cilia. To the best of our knowledge, this case represents one of the earliest reports to perform IF and ciliary movement analysis in PCD with a homozygous deletion involving exons 1–4 of *DRC1*.

## Graphical abstract

We report the case of a 29‐year‐old male diagnosed with primary ciliary dyskinesia (PCD) with a homozygous loss‐of‐function deletion involving exons 1–4 of *DRC1*. A high‐speed video microscopy analysis showed motile cilia with reduced amplitude, and immunofluorescence (IF) showed the absence of DRC3 in the cilia, suggesting a *DRC1* deletion affects DRC3 localization in the cilia. IF might be a potential tool to select patients with a *DRC1* deletion in the Japanese population.

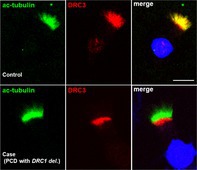

## 1 Introduction

Primary ciliary dyskinesia (PCD) is a genetically heterogeneous disorder of the ultrastructure and function of the cilia. A combination of investigations including transmission electron microscopy (TEM), high‐speed video microscopy analysis (HSVA), nasal nitric oxide (nNO) testing, immunofluorescence (IF), and genetic testing has been performed. Most PCD cases occur via autosomal recessive inheritance, and > 50 causal genes have been reported. DRC1 is the central subunit of the nexin‐dynein regulatory complex (N‐DRC), an axonemal structure critical for regulating ciliary motility, and *DRC1* mutations cause PCD [1]. More recently, a homozygous deletion involving exons 1–4 of *DRC1* has been found to be the most frequent in Japanese patients with PCD [2, 3]. Although genetic diagnosis is well established for a homozygous deletion involving exons 1–4 of *DRC1*, there are no reports on the usefulness of IF or the analysis of ciliary motility. Herein, we describe a patient previously diagnosed with refractory diffuse panbronchiolitis (DPB), who subsequently underwent PCD diagnostic examinations (genetic testing, TEM, HSVA, and IF of cilia). Genetic analysis confirmed a diagnosis of *DRC1*‐related PCD, and IF using the DRC3 antibody and ciliary movement analysis supported the diagnosis.

## 2 Case Report

**FIGURE 1:**
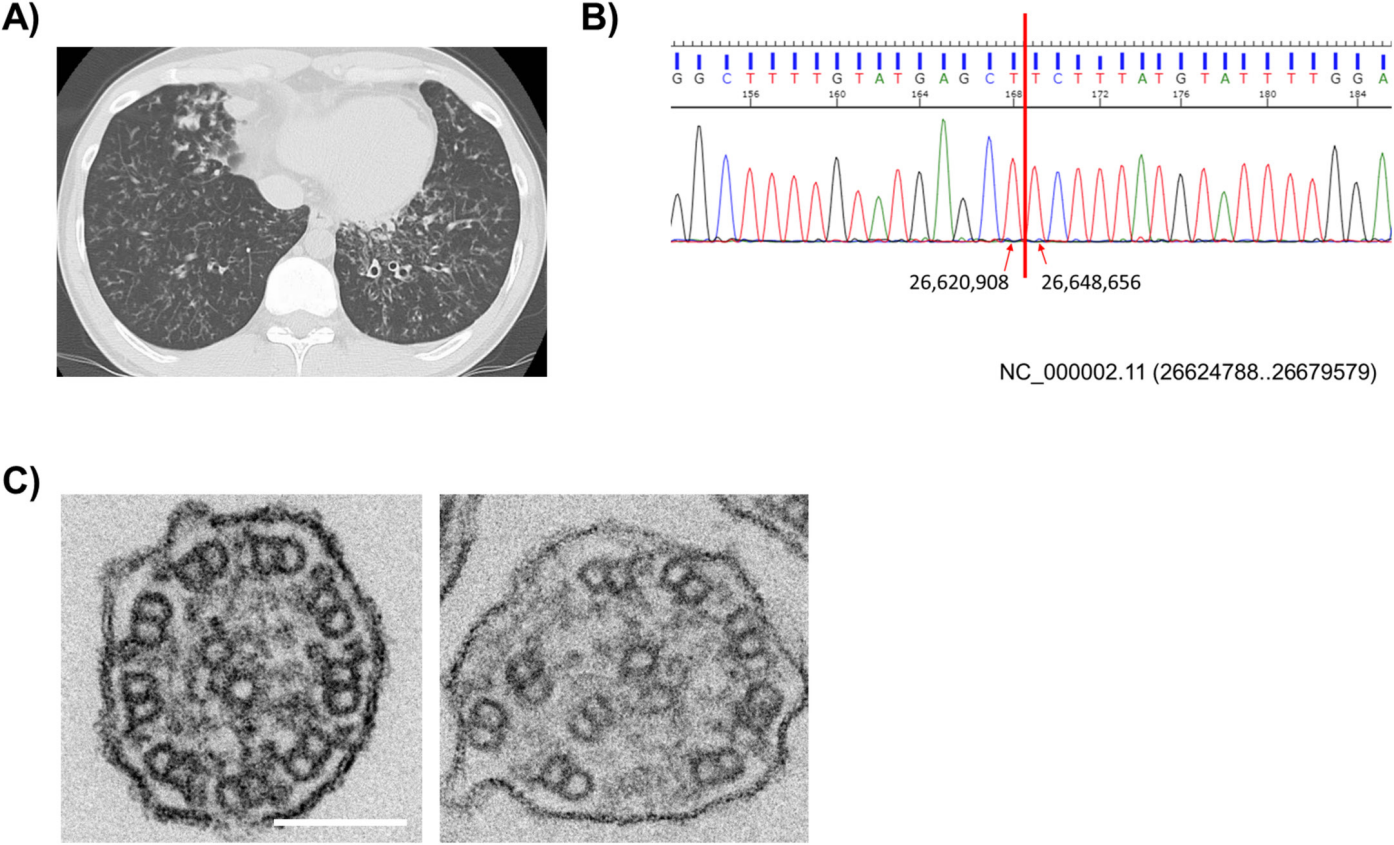
(A) Chest computed tomography (CT) image shows diffuse small granular opacities and bronchiectasis with mucus plugging. No situs abnormalities were identified. (B) Genetic analysis. A combination of the next‐generation sequencing panel and Sanger sequencing was performed. Green indicates adenine; black, guanine; red, thymine; and blue, cytosine. The genetic testing revealed a homozygous loss‐of‐function deletion involving exons 1–4 of *DRC1*. (C) Transmission electron microscopy (TEM). Most cilia revealed normal 9 + 2 microtubular arrangement and the presence of both inner and outer dynein arms (left) and some of the cilia with the transposition of peripheral microtubule pairs (right). The scale bar equals 100 nm.

**FIGURE 2:**
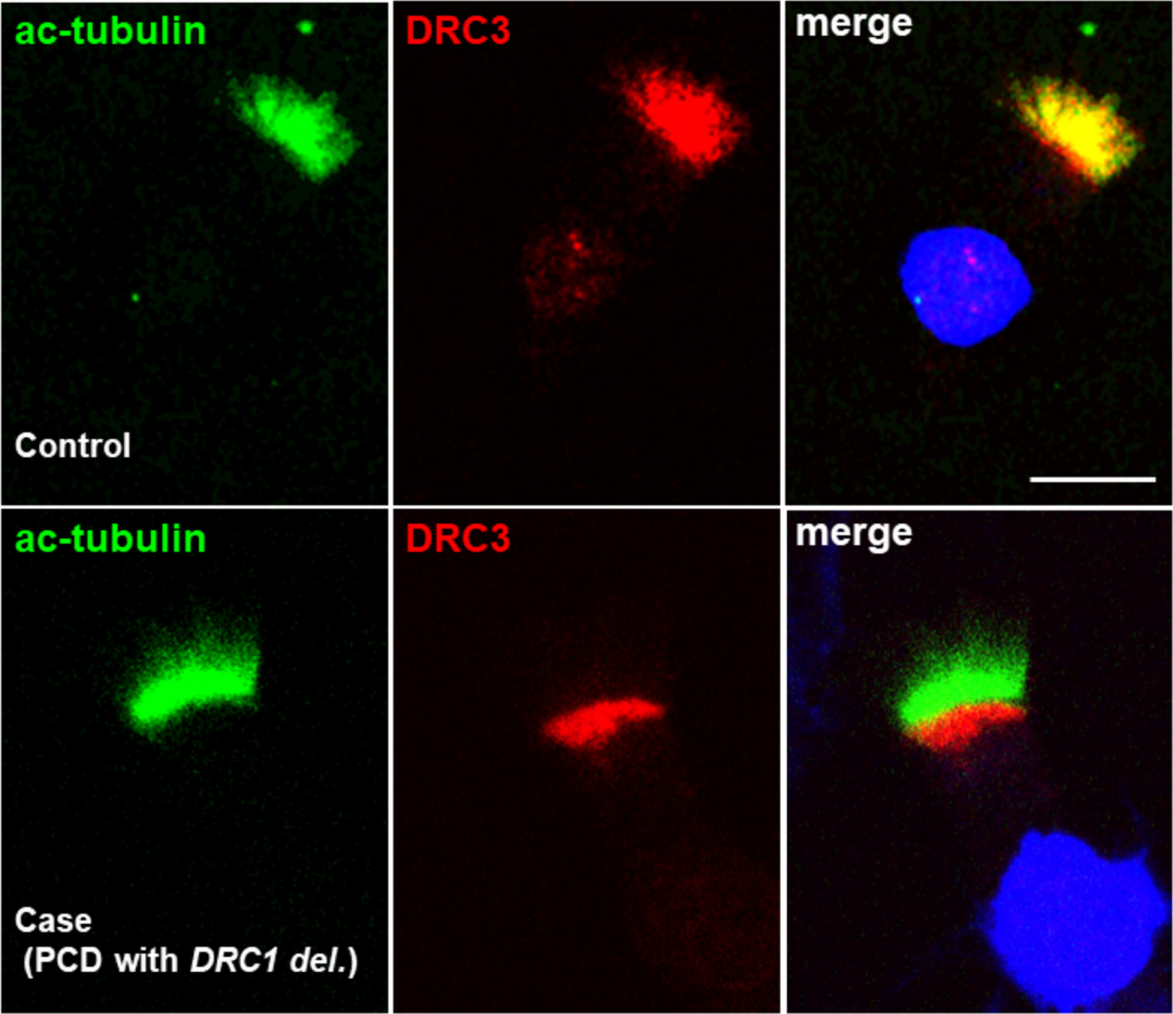
Immunofluorescence (IF) analysis of the cilia. In control cells, DRC3 was localised along the entire length of the axonemes. In contrast, patient samples exhibited DRC3 absence in the axonemes and mislocalization to the apical side of the cytoplasm. Anti‐DRC3 (HPA036040, Sigma‐Aldrich) (red) was counterstained with anti‐acetylated tubulin (T7451, Sigma‐Aldrich) (green) to visualise the entire ciliary axonemes. Merged images show the nucleus, which was visualised using 4′,6‐diamidino‐2‐phenylindole (DAPI) (blue). Yellow indicates colocalization and the presence of the protein of interest (DRC3) in the cilia. The white scale bars equal 10 μm. Ciliated cells collected from a patient highly unlikely to have PCD were used as control samples.

A 29‐year‐old male was referred to our hospital for PCD diagnostic examinations [4]. He presented with chronic productive cough and sinusitis since childhood. His parents are in a non‐consanguineous marriage, and his sister has been diagnosed with bronchiectasis. He was diagnosed with DPB 9 years prior to admission. Chest computed tomography (CT) revealed diffuse small granular opacities and bronchiectasis with mucus plugging. No situs abnormalities were observed (Figure 1A). His pulmonary function test results showed obstructive ventilatory impairment (FEV1 2.02 L (46.4% pred), FEV1/FVC 65.58%). He had a clinical history of resistance to long‐term treatment with macrolides and muscarinic antagonists. Genetic analysis of peripheral blood samples revealed a homozygous loss‐of‐function deletion involving exons 1–4 of *DRC1* (Figure 1B). This deletion of *DRC1* gene is expressed as NM_145038.4: c.1‐3952_540 + 1331del27748‐bp when the mRNA is used as a reference sequence [2]. This indicates that the deletion starts at 3952 bp upstream from the transcription start site. Since the deletion includes the promoter region biallelicly, this gene cannot possibly be transcribed. The patient underwent bronchoscopy for the analysis of cilia. Airway brushing samples were used for TEM and IF, and epithelial samples obtained from endobronchial forceps biopsies were used for HSVA. TEM revealed almost normal cilia and some cilia with the transposition of peripheral microtubule pairs (Figure 1C). HSVA of the original biopsy specimen revealed dyskinetic ciliary motion. To evaluate ciliary motion without the influence of secondary damage by inflammation or infection, we conducted an air‐liquid interface culture [4]. HSVA revealed decreased beat amplitude and somewhat stiff, dyskinetic waveforms compared to normal controls (Videos 1 and 2 [cultured cells in this case], 3 and 4 [normal controls]). The cultured cells from this case had a ciliary beat frequency (CBF) of 4.2 Hz, lower than the CBF of 8.6 Hz in normal controls. IF analysis was performed using antibodies against DRC3 (a protein in N‐DRC), DNAH5, and DNAH11 (proteins in the outer dynein arms). In this case, IF revealed the absence of DRC3 in the axonemes and its mislocalization to the apical side of the cytoplasm (Figure 2). DNAH5 and DNAH11 were expressed normally (data not shown).

**VIDEO 1:** High‐speed video microscopy analysis (HSVA) of cultured ciliated cells from this case with a homozygous deletion involving exons 1–4 of *DRC1*. Recorded from the top view. Video content can be viewed at https://onlinelibrary.wiley.com/doi/10.1002/rcr2.70424.

**VIDEO 2:** HSVA of cultured ciliated cells from this case with a homozygous deletion involving exons 1–4 of *DRC1*. Recorded from the side view. Video content can be viewed at https://onlinelibrary.wiley.com/doi/10.1002/rcr2.70424.

**VIDEO 3:** HSVA of cultured ciliated cells from normal controls. Recorded from the top view. Video content can be viewed at https://onlinelibrary.wiley.com/doi/10.1002/rcr2.70424.

**VIDEO 4:** HSVA of cultured ciliated cells from normal controls. Recorded from the side view. Video content can be viewed at https://onlinelibrary.wiley.com/doi/10.1002/rcr2.70424.

## 3 Discussion

In the Japanese population, a large deletion allele spanning 27,748 bp, including exons 1–4 of the *DRC1* gene, which encodes proteins in the N‐DRC, has a frequency of 0.2% in the general population, presumably as a founder mutation [3]. *DRC1* acts as a disease‐causing gene in 49% of Japanese PCD patients, followed by *DNAH5* (31%) and *DNAH11* (12%) [2]. In PCD with a *DRC1* deletion, TEM typically shows no prominent abnormalities: normal ciliary ultrastructure or an absence of nexin links [1], central apparatus abnormalities, or axonemal microtubular disorganisation [2]. In PCD with a *DRC1* mutation, ciliary motion shows a reduced bending amplitude [1]. PCD with a *DRC1* deletion may be misdiagnosed when relying solely on TEM or HSVA because of the absence of prominent abnormalities. The clinical features of PCD are similar to those of sinobronchial syndromes (SBS) including DPB. Keicho et al. reported a homozygous deletion involving exons 1–4 of *DRC1* in two (1.9%) of 105 Japanese adult patients clinically diagnosed with DPB in the past [3]. Situs inversus was not observed in any of the 21 Japanese patients with a *DRC1* deletion [2]. Therefore, it is believed that there are many undiagnosed cases of PCD among patients diagnosed with SBS or DPB in Japan. In this report, we present a case previously diagnosed with DPB. This case was diagnosed with PCD with a *DRC1* deletion identified by genetic testing, and TEM and HSVA using cultured cells revealed findings consistent with those of previous reports [1, 2, 3]. IF can detect and visualise major defects in the ciliary ultrastructure by using appropriate antibodies. In this study, because there is no commercially available DRC1 antibody verified for respiratory cilia, we used a DRC3 antibody for IF. DRC1, DRC2 and DRC4 are core N‐DRC structural components and serve as the scaffold for the assembly of DRC3, 5, 6, 7, 8, and 11 described by analyses of Chlamydomonas [5]. As demonstrated by IF analyses in Western literature [1], DRC3 proteins were absent from the cilia of PCD patients with *DRC1* mutations. In our case, IF similarly revealed the absence of DRC3 in the cilia. These findings indicate that *DRC1* mutations including a homogeneous deletion of exons 1–4 disrupt N‐DRC assembly, and result in DRC3 absence in the cilia. IF has proven useful for the detection of various PCD, including *DNAH5* and *DNAH11* mutations [3, 4], and has broad applicability in many research facilities. Therefore, IF with DRC3 antibodies might be a potential tool to select patients with a *DRC1* deletion in the Japanese population. Further studies with more cases are needed. To the best of our knowledge, this case represents one of the earliest reports to perform IF and ciliary movement analysis in PCD with a homozygous deletion involving exons 1–4 of *DRC1*.

## Author Contributions

A.K. and M.K. wrote the manuscript. M.K., K.T. and E.T. supervised the manuscript. M.T., Y.S., A.M., F.M., K.K., T.A., K.A. and O.Y. managed the patient. K.N., M.I. and K.T. conducted genetic analysis. All authors approved the final manuscript.

## Funding

This work was supported by JSPS Grants‐in‐Aid for Scientific Research (C) (Grant Numbers: 19K09886, 22K08244, 25K19448 and 25K12809) and (B) (Grant Number 25K02660) from the Ministry of Education, Culture, Sports, Science, and Technology of Japan and the Japan Agency for Medical Research and Development (AMED) (Grant Number: JP19ek0109410).

## Ethics Statement

PCD diagnostic examinations were approved by the Ethical Committee of Tokyo Women's Medical University (approval number: 258C; April 10, 2018, 5819; September 18, 2020).

## Consent

The authors declare that written informed consent was obtained for the publication of this manuscript and accompanying images using the consent form provided by the Journal.

## Conflicts of Interest

Keiko Kan‐o is Editor‐in‐Chief of the journal and co‐author of this article. She was excluded from the peer‐review process and all editorial decisions related to the acceptance and publication of this article. Peer review was handled independently by Deputy Editor Toshiaki Kikuchi to minimise bias. The other authors declare no conflicts of interest.

## Data Availability

The data that support the findings of this study are available from the corresponding author upon reasonable request.
